# Herpes Zoster Ophthalmicus in an Immunocompetent Child With Concurrent COVID-19 Infection: A Case Report and Literature Review

**DOI:** 10.7759/cureus.37020

**Published:** 2023-04-01

**Authors:** Hala El belidi, lalla ouafa cherkaoui

**Affiliations:** 1 Ophthalmology, Mohammed V University, Hospital of Specialties, Rabat, MAR

**Keywords:** pediatric, covid-19, varicella-zoster virus, herpes zoster, herpes zoster ophthalmicus

## Abstract

Herpes zoster ophthalmicus (HZO) is a rare complication of herpes zoster (HZ) that can occur in pediatric patients. It can have significant implications for affected individuals, with the potential for patients to experience ocular complications. Additionally, HZO can have a chronic disease course, requiring long-term treatment in some patients. Following the progression of the COVID-19 pandemic, reports worldwide have identified a potential association between HZO and COVID-19. This case report describes a rare case of a child presenting HZO during a COVID-19 infection.

## Introduction

Herpes zoster ophthalmicus (HZO) is a rare complication of herpes zoster (HZ) that can occur after primary varicella-zoster virus (VZV) infection or immunization. Herpes zoster is characterized by a distinct skin rash that typically follows a specific dermatomal pattern and does not cross the midline. It often arises due to a weakened cell-mediated immune system caused by factors such as illness, medication, or age [1,2].

In HZO cases, up to 50% of patients experience ocular complications. The underlying causes of these complications are not entirely clear, but they may be due to the reactivated varicella virus, a chronic active HZ infection, or an immune-mediated mechanism. Long-term treatment is required in approximately 20% of patients with HZO, as they experience a chronic disease course [3]. Although HZO is rare in childhood, with an annual incidence of 42 cases per 100,000 people, it can have significant implications for affected individuals [4].

Following the progression of the COVID-19 pandemic, reports worldwide have identified a potential association between HZO and COVID-19 [5]. Here, we report a rare case of a child presenting HZO during a COVID-19 infection.

## Case presentation

A five-year-old male child presented to the emergency department of ophthalmology with a complaint of a tingling and itching sensation on the right side of his face, which persisted for two days, followed by a vesicular skin eruption on the eyelids. The child was diagnosed with mild to moderate COVID-19 five days prior, and he received supportive and symptomatic treatment. Furthermore, the patient reported a previous episode of chickenpox at the age of three years, and he had not been vaccinated against the varicella-zoster virus. The child had no significant medical history other than this.

**Figure 1:**
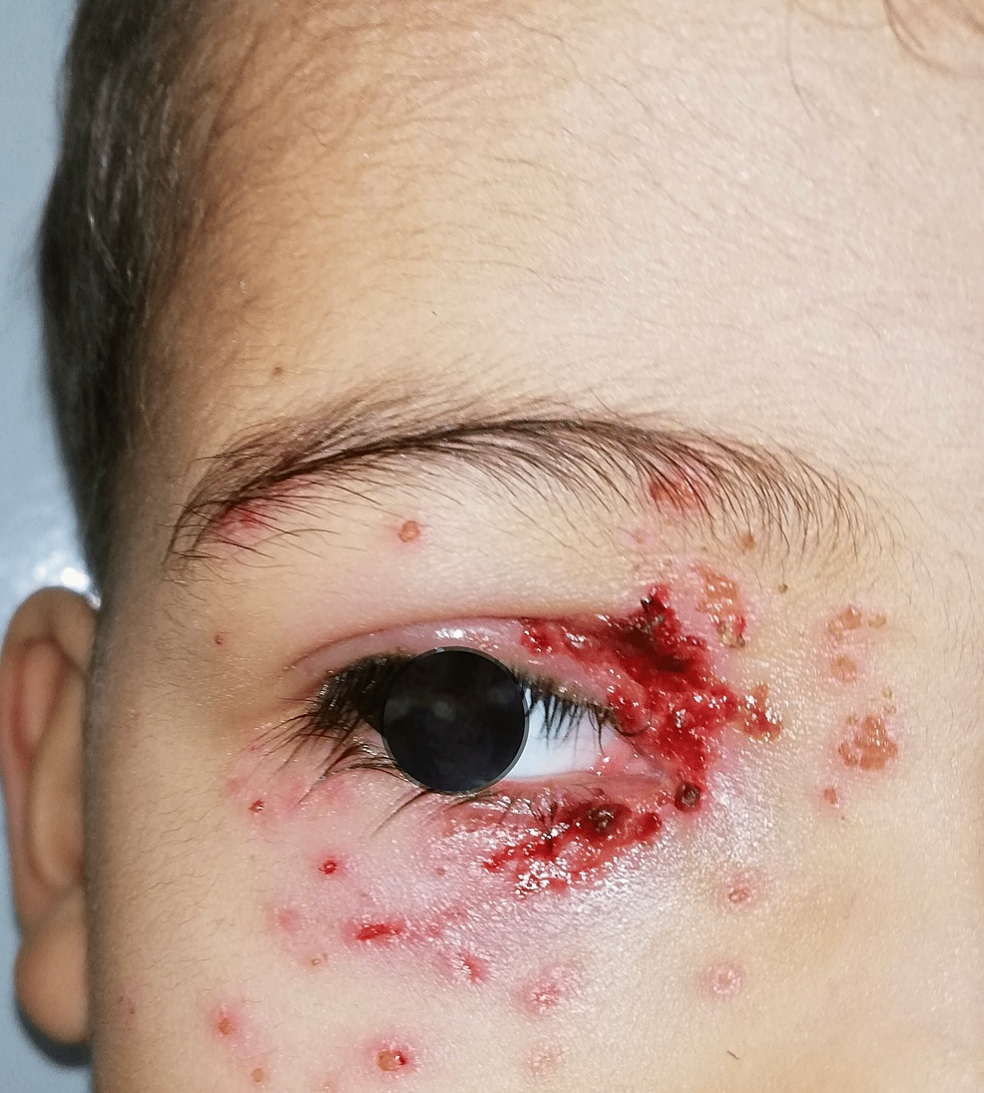
Clinical presentation of the patient showing Hutchinson's sign

**Figure 2:**
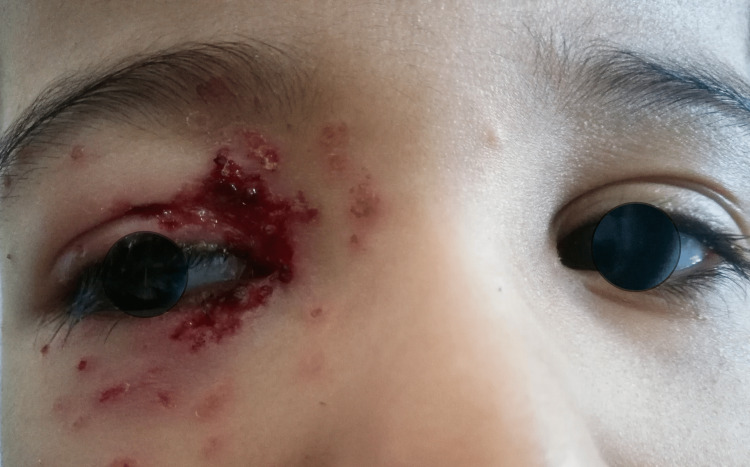
Vesicular papulopustular skin lesions on the right side of the face, not crossing the midline

During the ophthalmological examination, crusted vesicular lesions were observed on the right eyebrow and eyelids, extending up to the root of the nose (Figures 1, 2). Visual acuity was preserved at 10/10 in both eyes. Examination of the right eye using a slit lamp showed some conjunctival secretions without hyperemia, and the cornea was clear with a quiet anterior chamber. The posterior segment examination results were normal. The left eye examination was unremarkable. The child's vital signs were normal, and general examination revealed no anomalies, including the absence of meningeal irritation. The child was diagnosed with HZO, and treatment was initiated with systemic and topical acyclovir for seven days, which resulted in the complete resolution of the skin rash. Routine blood tests showed no abnormalities, and the child underwent regular ophthalmological examinations thereafter. One year later, the child's ocular examination remains normal.

## Discussion

In the realm of pediatric medicine, HZ is considered a rare occurrence, with an incidence ranging from 0.2 to 0.75 cases per 1000 person-years [6]. In countries with prevalent VZV immunization, the incidence of HZ in children is relatively low, at approximately one case per 1000 person-years. However, despite vaccination, VZV latency can still be established [3]. According to a survey that involved 322 children with HZ, the incidence rate differed by 79% between vaccinated and unvaccinated subjects [7]. Patients who are at a higher risk of developing HZO are those who have primary or secondary immunodeficiency, contracted primary varicella infection during the first year of life, or have had intrauterine varicella exposure [3]. However, in a comprehensive evaluation of pediatric HZ hospitalizations across Germany, it was found that among the 244 children studied, 59% had no underlying immunodeficiency [8]. Interestingly, HZO was observed in 12% of children with HZ and was more prevalent in immunocompetent children (27%) than in immunocompromised children (16%) (p=0.39).

Herpes zoster ophthalmicus is a rare condition for which randomized controlled trials are lacking. Therefore, some of the data on its management is extrapolated from herpes simplex virus (HSV) disease due to the similarities in latency and reactivation patterns of the two viruses. According to Kennedy et al., VZV establishes latency by retrograde axonal transport or infected memory T-cells via a process called "round-trip infection" [9]. The virus replicates in the neuron, travels to the skin, and produces new lesions before returning to the ganglia to infect more neurons. Although stromal keratitis or uveitis in HZO may not be due to active infection, subclinical or intermittent viral shedding may be the cause of subsequent inflammation.

Diagnosis of HZO is typically made based on clinical presentation, but the polymerase chain reaction (PCR) technique can rapidly confirm the diagnosis, especially in immunocompromised individuals. Acyclovir is the recommended first-line treatment for children, with a suggested dose of 10 mg/kg every eight hours. Starting treatment within 72 hours of initial symptoms significantly reduces adverse outcomes. Topical steroids are recommended for the treatment of conjunctival, corneal, and uveal inflammation [10].

There is currently no consensus on the duration of antiviral treatment or the need for secondary prophylaxis in pediatric HZO. The German Dermatology Society suggests maintaining a sufficient virostatic plasma level with high-dose acyclovir or valacyclovir and highlights the need for longer treatment duration in certain cases [11]. They also recommend balancing the antiviral effect and the tissue-damaging immune reaction when using systemic combination treatment with acyclovir and prednisolone.

It is uncommon for children to develop HZO, despite its high prevalence in adults. The onset of HZO in children is sudden and accompanied by severe systemic symptoms; however, the disease typically has a mild course, with complete resolution within several weeks. Although scarring can occur in some cases, visual impairment is not common [12].

In our case, the patient was diagnosed with HZO while also infected with COVID-19. Given the lack of any predisposing factors for VZV reactivation, it appears that the acute illness and the associated physical and emotional stress of COVID-19 may have been the triggering factor for the development of HZO.

Over the past two years of the COVID-19 pandemic, there have been several cases of co-infections and superinfections occurring alongside COVID-19 [13]. Among patients with COVID-19, various skin manifestations have been reported, including HZ [14]. As far as we know, HZO has only been documented in six COVID-19 patients so far: two children and four young adults. It's worth noting that all patients had a previous history of varicella, and only one young patient had an immunodeficiency [15-17].

## Conclusions

Herpes zoster ophthalmicus is a rare but serious complication of HZ infection that can affect the eyes, and up to 50% of patients experience ocular complications. Diagnosis of HZO is typically based on clinical presentation, but PCR can be used to confirm the diagnosis rapidly, especially in immunocompromised individuals. Antiviral treatment with acyclovir is recommended, and topical steroids may be used for the treatment of conjunctival, corneal, and uveal inflammation. There is currently no consensus on the duration of antiviral treatment or the need for secondary prophylaxis in pediatric HZO. It is essential to recognize the association between HZO and COVID-19 to manage affected individuals adequately.
